# Effect of Surfactants on the Microstructures of Hierarchical SnO_2_ Blooming Nanoflowers and their Gas-Sensing Properties

**DOI:** 10.1186/s11671-018-2656-5

**Published:** 2018-08-22

**Authors:** Yan-Fei Zhao, Yu-Ping Sun, Xiu Yin, Guang-Chao Yin, Xiao-Mei Wang, Fu-Chao Jia, Bo Liu

**Affiliations:** 1 Laboratory of Functional Molecules and Materials, School of Physics and Optoelectronic Engineering, Shandong University of Technology, Zibo, 255000 China; 2 School of Materials Science and Engineering, Shandong University of Technology, Zibo, 255000 China

**Keywords:** Tin dioxide, Gas sensor, Surfactant, Nanoflowers

## Abstract

Hierarchical SnO_2_ blooming nanoflowers were successfully fabricated via a simple yet facile hydrothermal method with the help of different surfactants. Here we focus on exploring the promotion effects of surfactants on the self-assembly of 2D SnO_2_ nanosheets into 3D SnO_2_ flower-like structures as well as their gas-sensing performances. The polyporous flower-like SnO_2_ sensor exhibits excellent gas-sensing performances to ethanol and H_2_S gas due to high porosity when polyvinyl pyrrolidone is added into the precursor solution as a surfactant. The response/recovery times were about 5 s/8 s for 100 ppm ethanol and 4 s/20 s for 100 ppm H_2_S, respectively. Especially, the maximum response value of H_2_S is estimated to be 368 at 180 °C, which is one or two orders of magnitude higher than that of other test gases in this study. That indicates that the sensor fabricated with the help of polyvinyl pyrrolidone has good selectivity to H_2_S.

## Background

Gas sensors have attracted a widespread attention due to their potential applications in detecting toxic, noxious, flammable, and explosive gas [1]. At present, the metal oxide semiconductors occupy an important position in various sensors due to their simple preparation process, lower cost, and higher sensitivity to the target gases [2–4]. Tin dioxide (SnO_2_), a multifunctional n-type material with a direct band gap of 3.6 eV [5], has been widely used in both fundamental study and practical applications, such as gas sensors [6], catalysis [7], and optoelectronic devices [8]. Especially, SnO_2_ has been regarded as the most potential sensing material owing to its natural non-stoichiometry [9], high sensitivity, fast response/recovery speed, and high chemical stability [10].

It is well known that the gas-sensing mechanism of metal oxides is related to the adsorption and desorption processes of the target gas on the sensor surface, giving rise to a change of the electrical conductivity [11]. These processes strongly depend on the size, morphology, and dimension as well as crystalline structure of the samples [12]. There are two main ways to effectively enhance the sensing performance of SnO_2_ [13]. One is to synthesize composed materials based on SnO_2_, such as fabrication of p-n junctions, surface decoration, or doping [14]. The other is to prepare various pure SnO_2_ materials including nanotubes [15], nanorods [16], nanospheres [17], hollow structures [14], and nanoflowers [18], which have unique nanostructures, high specific surface area, and strong electron capture abilities [19]. Recently, three-dimensional (3D) hierarchical SnO_2_ nanostructures have drawn much attention because of their better gas-sensing performance caused by large specific surface area and rapid gaseous diffusion compared with 1D and 2D nanostructures [20]. Various techniques have been used to fabricate 3D nanostructures of SnO_2_ [21], such as chemical vapor deposition [22], solvothermal synthetic method [23], template method [24], sol–gel method [25], and hydrothermal route [26]. Among them, solvothermal and hydrothermal routes with low cost [27], high yields, and simple manipulation have been proved to be the promising methods to synthesize 3D hierarchical SnO_2_ nanostructures. For instance, Dong et al. prepared hollow SnO_2_ nanospheres with the diameter ranging from 200 to 400 nm using a solvothermal synthetic method [28]. Li et al. fabricated a novel snowflake-like SnO_2_ hierarchical architecture with excellent gas-sensing properties via a facile hydrothermal method [29]. Moreover, Chen et al. successfully synthesized hierarchical flower-like SnO_2_ blooming nanoflowers constructed by self-assembly of many regular-shaped nanosheets through conventional hydrothermal method [30].

The practical application of SnO_2_ sensors is still limited to a certain extent due to the relative higher working temperature and poorer selectivity to test gases [31]. In order to improve the gas-sensing properties, researchers have paid attention to controllable synthesis of 3D flower-like SnO_2_ nanostructures with surfactant effects [32], yet a significant challenge is posed because of the variety of surfactants.

In the present study, we report a well-controlled optimization of 3D hierarchical SnO_2_ nanoflowers based on self-assembly of thin nanosheets with the help of different surfactants under hydrothermal condition. Our systematically comparative gas-sensing study between fabricated sensors focuses on the promotion effect of surfactants on sensor behaviors. The results show that the amphiphilic non-ionic surfactants, such as PVP and Triton X-100, can be potential candidates to optimize the morphology of 3D nanoflowers with high porosity and large specific surface area. Particularly, the sensor based on PVP exhibits high response, fast response time, and good selectivity to H_2_S at a relative lower temperature. In addition, a possible well-controlled growth mechanism of SnO_2_ nanostructures is proposed.

## Methods/Experimental

Trisodium citrate dihydrate and tin chloride dihydrate from Sinopharm Chemical Reagent Co., Ltd. were used as precursors for SnO_2_ synthesis. Polyethyleneimine, hexamethylene tetramine, TritonX-100, and polyvinylpyrrolidone were purchased from Aldrich Chemistry and used as the structure-directing agents. Distilled water was used throughout the experiments. All chemicals were of analytical grade and used as-purchased without any further purification.

### Synthesis of SnO_2_ Nanoflowers with Different Architectures

**Fig. 1:**
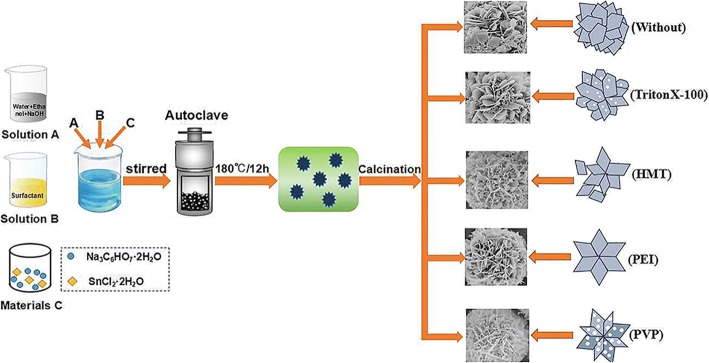
Schematic illustration of the formation process of the hierarchical flower-like SnO_2_ nanostructures using different kinds of surfactants

A typical synthesis procedure with simple hydrothermal method can be described as follows (Fig. 1): firstly, 5 mmol of NaOH was added into an 80-ml mixture of anhydrous ethanol and deionized water (1:1) under magnetic stirring. Then, 20 mmol Na_3_C_6_H_5_O_7_·2H_2_O and 10 mmol SnCl_2_·2H_2_O were dissolved into the mixed solution successively under vigorous stirring for 1 h at room temperature. The mixed solution was then transferred into a 100-mL Teflon-lined stainless steel autoclave, and maintained at 180 °C for 12 h, and then cooled down to room temperature naturally. After reaction, the obtained precipitate was collected by centrifugation, washing with deionized water and anhydrous ethanol for several times, and dried at 60 °C for 6 h. The SnO_2_ nanoflowers were finally obtained after calcining the precipitate in a muffle furnace under air ambient condition at 500 °C for 2 h. In order to synthesize SnO_2_ nanoflowers with different microstructures, different surface active agents (1.0 g) were introduced into the solution respectively before the dissolution of Na_3_C_6_H_5_O_7_·2H_2_O. In this work, four different kinds of surfactants were used, including PVP, PEI, HMT, and TritonX-100, and the corresponding final products are named as S_PVP_, S_PEI_, S_HMT_, and S_TritonX-100_, respectively, while the product without surfactant is signed as S_0_.

### Characterizations

It is well known that the gas-sensing properties of gas sensors are highly related to the morphology, size, and dispersibility of nanomaterials. The as-prepared products were analyzed in terms of their structures and morphologies by means of polycrystalline X-ray diffraction (XRD, Germany Bruker AXS D8 Advance), scanning electron microscopy (SEM, USA FEI Sirion 200), and field emission transmission electron microscopy (FETEM, USA Tecnai G2 F20 S-TWIN). The surface area is measured using Elemental Analyzer (USA ASAP 2460) based on the Brunauer-Emmett-Teller (BET) method.

### Sensor Fabrication and Gas-Sensing Test

**Fig. 2:**
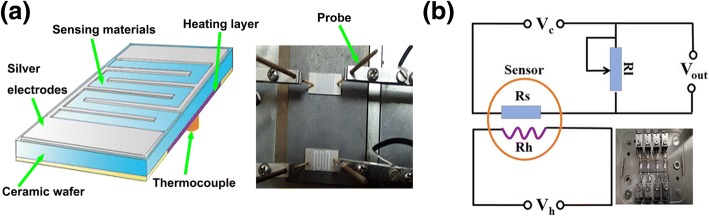
**a** Schematic diagram of the gas sensor configuration. **b** Electric circuit diagram of sensor device

The gas sensor was fabricated using screen printing method on top of an alumina tube (seen in Fig. 2a). Typically, a proper amount of as-prepared powder was firstly mixed with anhydrous ethanol to form a slurry suspension. Subsequently, the slurry suspension was coated onto the alumina tube by a small brush, which is supported by two Au electrodes and four Pt conducting wires. Next, a Ni-Cr heating wire was inserted into the alumina tube to control working temperature by tuning the heating voltage. Finally, the product was aged at 80 °C for 72 h before testing.

The gas-sensing properties were measured using chemical gas sensor-4 temperature pressure small (CGS-4TPs) intelligent gas-sensing analysis system (Beijing Elliott Technology Co., Ltd., China) under the lab conditions. Figure 2b displays the typical schematic electrical circuit. R_s_ is the resistance of sensor and R_l_ is the load resistance, and a heating voltage (V_h_) is used to adjust the working temperature. In the present work, the response of the sensor was defined as S = (R_s_ − R_g_)/R_g_, where Rs is the initial resistance and Rg is the resistance after injection of gases. The response and recovery times are defined as the time taken by the sensor to achieve 90% of the total resistance change in the case of adsorption and desorption, respectively.

## Results and Discussion

### Structural and Morphological Characterization

**Fig. 3:**
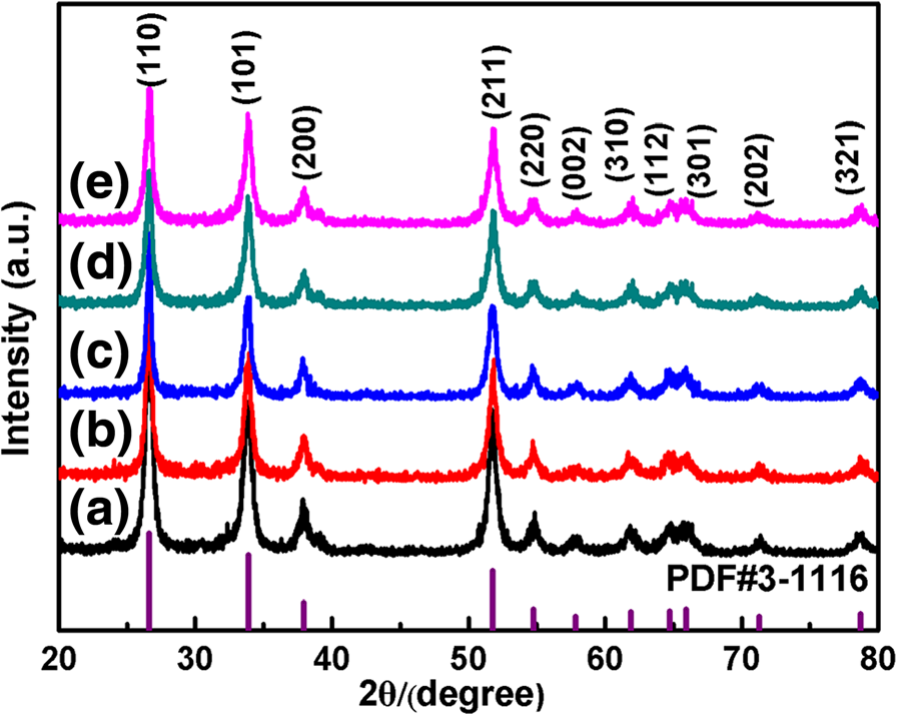
XRD patterns of the SnO_2_ samples with different morphologies. **a** S_0_, **b** S_TritonX100_, **c** S_HMT_, **d** S_PEI_, and **e** S_PVP_

The crystalline phase of the as-prepared SnO_2_ products was identified by power X-ray diffraction as shown in Fig. 3. From the XRD pattern, all the observed diffraction peaks can be easily assigned to the tetragonal rutile structure of pure SnO_2_ with the standard JCPDS file card no. 41-1445, and no other peaks can be identified due to impurities. The sharp peaks indicate the high degree of crystallinity of our SnO_2_ samples, and no remarkable shift is detected in the diffraction peaks, revealing that the samples are of high purity.

**Fig. 4:**
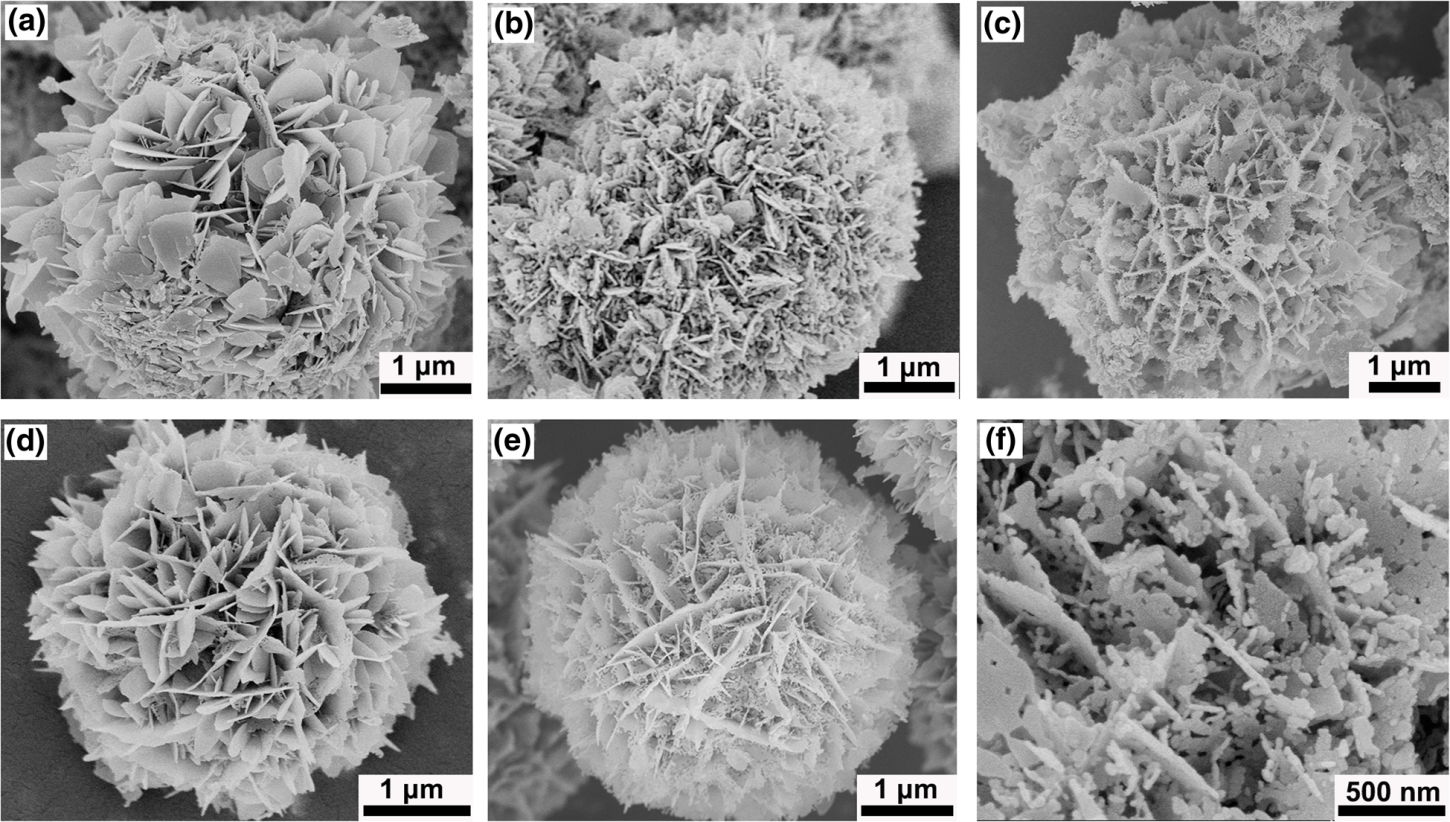
The SEM images of the SnO_2_ nanoflowers with different morphologies. **a** S_0_, **b** S_TritonX-100_, **c** S_HMT_, **d** S_PEI_, and **e**, **f** S_PVP_

Figure 4a shows the SEM image of the product without surfactant. A hierarchical flower-like architecture can be observed and the unique nanoflowers are assembled by ultrathin nanosheets with an average thickness of 20 nm around. Unfortunately, these nanosheets are closely staggered with respect to each other, which results in a sharp decrease in its reaction spaces. Figure 4b–e shows the morphologies of the products obtained by introducing different surface active agents while keeping other experimental conditions unchanged. One can see that upon addition of TritonX-100 surfactant (Fig. 4b), the nanosheets are loosely intersected with each other, and some mesopores are shaped at the edge of the nanosheets. When HMT was added into the reaction mixture as the surface active agents (Fig. 4c), it can be seen that the nanosheets are arranged randomly and a number of smaller nanosheets are formed between ultrathin nanosheets. Figure 4d shows the SEM images of the products obtained by introducing PEI surfactant in the precursor solution, which reveals that the nanosheets with smooth surfaces are arranged orderly and are vertically intersected with each other, leaving a larger reaction space. Figure 4e, f presents the typical SEM images of the products obtained upon addition of PVP surfactant under the same conditions. One can see that the nanosheets are uniformly distributed along the radius across the whole sample to form a flower-like structure. Moreover, compared with other structures of S_TritonX-100_, S_HMT_, and S_PEI_, the nanosheets of S_PVP_ are enclosed into an inverted triangle cone with a relative larger hollow space (Fig. 4e). Further enlarged image reveals that the flower-like architectures are assembled by mesoporous nanosheets to form an open porous hierarchical structure, and each nanosheet was fabricated with numerous mesopores (Fig. 4f).

**Fig. 5:**
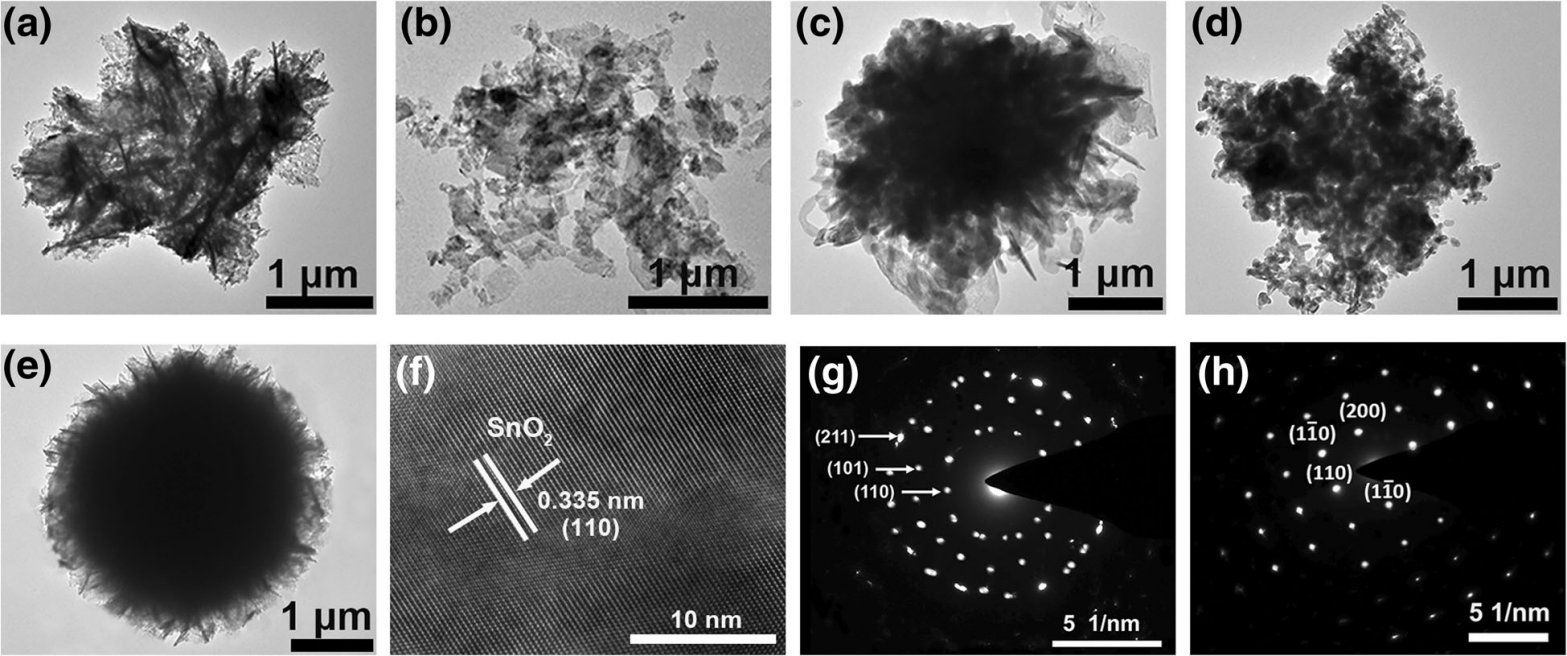
Low-magnification TEM images of SnO_2_ samples. **a** S_0_, **b** S_TritonX-100_, **c** S_HMT_, **d** S_PEI_, and **e** S_PVP_. **f** HRTEM micrograph showing enlarged lattice images of S_HMT_. **g** SAED patterns of S_0_. **h** SAED patterns of S_PVP_

In order to further investigate the microstructures and crystalline properties of the nanoflowers, low-magnification TEM and typical HRTEM combined with the selected area electron diffraction (SAED) analysis techniques are employed. From TEM images (Fig. 5a–e), it can be seen that the nanoflowers with an average diameter of 3 μm are assembled of numerous individual nanosheets, and its morphology and size are similar to the SEM images. Especially, the TEM image of S_PVP_ (Fig. 5e) shows that the most flower-like structure with the uniform dark color in the middle region is constructed from the well dispersion of numerous uniform nanosheets along the radius direction. Combining the SEM with TEM measurements, a conclusion can be drawn that the structures obtained upon the addition of PVP surfactant are the most stable. The high-resolution TEM (HRTEM) images show that for samples S_0_, S_HMT_, S_PEI_, and S_PVP_, the observed 0.335-nm lattice spacing is consistent with the (110) lattice plane of tetragonal rutile SnO_2_ (Fig. 5f only shows a typical HRTEM image for S_HMT_ as a representative). The exposure of (110) lattice plane reveals that (110) lattice plane is the most stable plane for SnO_2_ in air, which is consistent with the theoretical study. It should be noted that S_TritonX-100_ is a special case in this work (Fig. 5b). Upon addition of TritonX-100 surfactant, the growing and the dispersion of nanosheets are randomly leading to a relative larger diameter (3~4 μm ) of the nanoflowers compared with other samples. Moreover, its HRTEM image shows that the calculated lattice spacing is 0.264 nm, which is corresponding to (101) lattice plane of tetragonal rutile structure of SnO_2_. Furthermore, SAED pattern reveals that S_PVP_ has a nearly perfect single crystalline structure, and the diffraction spots correspond to (110),($1\;\overline{1}\;0$), ($\overline{1}\;1\;0$), and (200) lattice planes of SnO_2_ (Fig. 5h). Contrarily, for other samples such as S_0_, S_HMT_, S_PEI_, and S_TritonX-100_, the SAED pattern shows a polycrystalline structure, and the diffraction ring is indexed to the (110), (101), and (211) planes of tetragonal rutile structure of SnO_2_ (Fig. 5g).

### Growth Mechanism of the SnO_2_ Nanoflowers

Based on the experimental observations and analysis above, it is believed that the surfactants play a significant role in the formation of various SnO_2_ nanoflowers [33]. The possible growth mechanism of hierarchical sheet-flower SnO_2_ nanostructures is briefly illustrated in Fig. 1. In this work, all SnO_2_ nanoflowers are synthesized using SnCl_2_ as precursor [34]. Under the hydrothermal conditions, the overall reaction for the growth of SnO_2_ crystals with high temperature and pressure can be expressed as follows [35]:

1 $$ {\mathrm{SnCl}}_2+2{\mathrm{OH}}^{-}\to \mathrm{Sn}{\left(\mathrm{OH}\right)}_2+2{\mathrm{Cl}}^{-} $$

2 $$ \mathrm{Sn}{\left(\mathrm{OH}\right)}_2\to \mathrm{Sn}\mathrm{O}+{\mathrm{H}}_2\mathrm{O} $$

3 $$ \mathrm{SnO}+\frac{1}{2}{\mathrm{O}}_2\to {\mathrm{SnO}}_2 $$

4 $$ \mathrm{Sn}{\left(\mathrm{OH}\right)}_2+\frac{1}{2}{\mathrm{O}}_2+{\mathrm{H}}_2\mathrm{O}\to \mathrm{Sn}{\left(\mathrm{OH}\right)}_4\to {\mathrm{SnO}}_2+2{\mathrm{H}}_2\mathrm{O} $$

During the whole process, three chemicals greatly affect the morphology growth of SnO_2_ nanoflowers, including NaOH, sodium citrate, and the surfactant. Firstly, a number of tiny primary nanocrystals were formed due to the hydrolysis of Sn^2+^ in a basic ethanol-water solution as well as its rapid reaction with OH^−^ ions from NaOH. It should be noted that the basic ethanol-water environment is significant for stimulating the SnO_2_ nucleation and growth [36]. The addition of sodium citrate plays a crucial role in the space distribution of precursors due to its strong coordinating ability, which can promote an anisotropy in the fast growth and aggregation of SnO_2_ nanosheets with the driving force of decreasing surface energy and accelerate the assembling of nanosheets into the stable hierarchical blooming nanoflowers [37].

Generally, the addition of surfactants is favorable for the enlargement of the surface area as well as the enhancement of the surface activity [38]. Among the surfactants used in this work, PEI is one kind of cationic surfactants. When PEI is added into the reaction solutions, due to the existent N^+^ ions with a hydrophilic tail, PEI will preferentially adsorb on a certain crystal facet, which is conducive to the nucleation of SnO_2_ nanocrystals as well as the orderly growth of SnO_2_ nanosheets with a directional selectivity. Both PVP and TritonX-100 are amphiphilic non-ionic surfactants, which can serve as a soft template in the fabrication of mesoporous materials. Let us take PVP for example to explain the growth mechanism of porous structures on the SnO_2_ nanosheets as follows: when PVP is added into the solution, PVP molecules self-assemble into spherical micelles because of the strong hydrophobic attraction between the straight alkyl tails. Due to its amphiphilicity, the hydrophilic radical will move toward the direction of aqueous solution, and the hydrophobic radical will move in the opposite direction, leading to the formation of inorganic domains around periodically arranged PVP micelles. Then, Sn^2+^ and OH^−^ ions are easily adsorbed on the outer surfaces of these micelles via electrostatic interactions until SnCl_2_ is oxidized into SnO_2_ nanosheet, which are followed by the self-assembly of nanosheets into blooming nanoflowers with the help of sodium citrate. Finally, the soft-template PVP micelles are removed during the calcination process, yielding hierarchical SnO_2_ nanoflowers with mesoporous structures. Although both PVP and Triton X-100 contributed to the formation of porous structures, it should be noted that PVP can also play a role of dispersing agent, which makes the SnO_2_ nanosheets grow more uniformly and separately owing to the strong interactions and short electrostatic interaction distances between the SnO_2_ nanosheets and PVP.

### Gas-Sensing Properties

As reported previously, the hierarchical flower-like nanostructures were favorable for the absorption and diffusion of probe gases in sensor materials. To shed light on the promotion effect of surfactants and corresponding morphology on sensor behavior, a systematically comparative gas-sensing study between fabricated sensors is performed in this work.

#### Gas-Sensing Behaviors of Fabricated Sensors to Ethanol

**Fig. 6:**
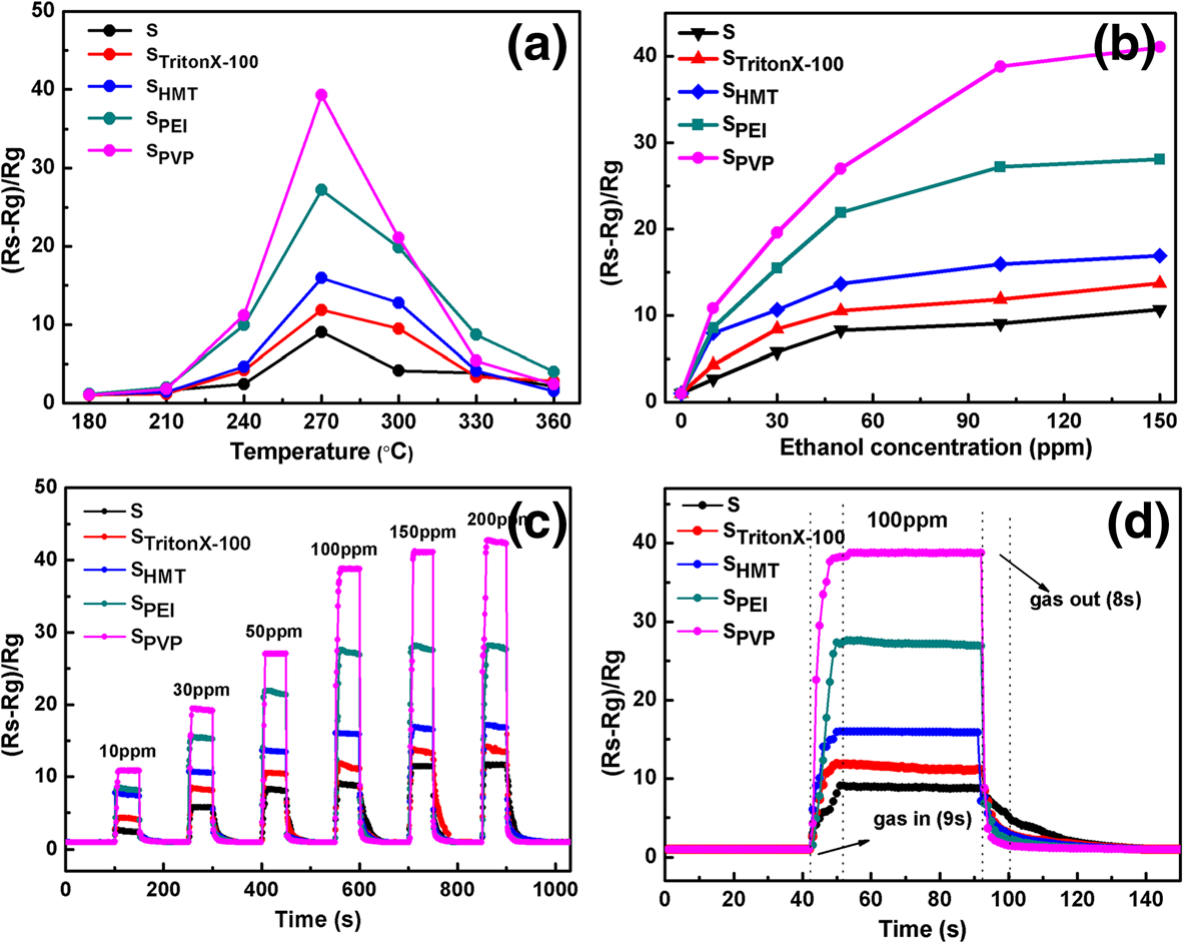
**a** The response of the sensors to 100 ppm ethanol under different operating temperatures (180–360 °C). **b** The dynamic response curves of the sensors to ethanol with different concentrations (10–150 ppm) at 270 °C. **c** Response versus time curves of the sensors to 10–200 ppm ethanol consecutively at 270 °C. **d** Dynamic sensing transient of the sensors to 100 ppm ethanol at 270 °C

The optimum operating temperature is a key factor for the application of semiconductor oxide gas sensors. Firstly, the responses of sensors to 100 ppm ethanol gas at various operating temperatures from 180 to 360 °C are tested as shown in Fig. 6a. It is clearly observed that all these sensors exhibit a similar gas-sensing behavior, i.e., the response values first increase with a rise of temperature, reach a maximum value at 270 °C, and then decrease gradually with a further increasing of temperature. Therefore, 270 °C can be chosen as the optimized operating temperature for gas-sensing study of all fabricated flower-like SnO_2_ sensors in our work. The reason for the dependence of the response on the temperature is as follows: When the operating temperature is too low, a relative smaller response value is assigned to the inert response due to chemical activation, while for too high operating temperature, the absorbed gas target molecules can escape from the sensors before reactions, resulting in a poor response as well. In addition, it can be seen from Fig. 6a that of all the five SnO_2_ sensors based on different surfactants, S_PVP_ shows the highest response to ethanol gas and the largest gas response value (38). The maximum response values of other four sensors are 27 for S_PEI_, 16 for S_HMT_, 11 for S_TritonX-100_, and 8 for S_0_.

Figure 6b shows the response of all SnO_2_ sensors toward ethanol in the concentration range 10~150 ppm at the optimal work temperature 270 °C. It can be clearly observed that the response of all sensors increases rapidly with the gas concentration below 50 ppm, and this trend becomes smooth from 50 to 150 ppm, tending to be saturated at about 100 ppm. As expected, the surfactants and the induced morphologies can produce a large influence on the gas-sensing of fabricated sensors. Among these fabricated sensors, the S_PVP_ sensor exhibits the best sensing behavior toward ethanol gas, and S_PEI_ comes second. To get a deep insight of gas-sensing mechanism, BET (Brunaure-Emmett-Teller) nitrogen adsorption-desorption is also performed to determine the specific surface areas of these samples, as shown in Table 1. One can see that S_PEI_ has the largest specific surface area (38.4 m^2^ g^−1^) with overall majority. It is noteworthy that despite the relative smaller surface area (15.5 m^2^ g^−1^), S_PVP_ is the best candidate for ethanol gas sensor due to its perfect flower-like architecture with orderly stacking self-assembly and relative higher porosity, providing more active adsorption sites for ethanol molecules. Even at low ethanol concentration as 10 ppm, the sensitivity of S_0_, S_TritonX-100_, S_HMT_, S_PEI_, and S_PVP_ sensors can reach 2, 4, 7, 9, and 11, respectively, indicating their potential application for ethanol sensors even at low concentrations.

**Table 1:** The BET for the prepared samples

Morphology	S_0_	S_TritonX-100_	S_HMT_	S_PEI_	S_PVP_
BET	14.5 m^2^ g^−1^	14.6 m^2^ g^−1^	15.2 m^2^ g^−1^	38.4 m^2^ g^−1^	15.5 m^2^ g^−1^

Figure 6c displays dynamic gas-sensing response and recovery curves of fabricated sensors toward ethanol with an operating temperature of 270 °C, from which one can see that the responses of all fabricated sensors increase with increasing the ethanol concentration, and a remarkable modulation of resistance is achieved at about 100 ppm. The responses show a drastic rise once the sensor was exposed to target gases and then dropped to its initial value in air. As shown in Fig. 6d, the response and recovery time to 100 ppm ethanol are about 16 s and 28 s for S_0_, 14 s and 18 s for S_TritonX-100_, 11 s and 15 s for S_HMT_, 9 s and 11 s for S_PEI_, and 5 s and 8 s for S_PVP_, respectively. It is obvious that the S_PVP_ sensor has the best response/recovery characteristics compared with other sensors.

Table 2 shows a comparison of ethanol-sensing performances based on different SnO_2_ fabricated approaches reported in other literatures and this work at the concentration of 100 ppm. One can see that our polyporous SnO_2_ nanoflower presents remarkably ethanol-sensing behaviors with lower optimal operating temperature and higher response value as well as faster response-recovery time, which could be attributed to the presence of numerous mesopores in SPVP sensor, leading to a high porosity in favor of the adsorption and diffusion of ethanol gas.

**Table 2:** The comparison of ethanol sensing performances of SnO_2_ materials based on different fabricated approaches reported in other literatures and this work

Materials	Synthetic methods	Working temperature (°C)	Sensor response	Response/recovery time (s)	Reference
SnO_2_ nanoparticles	Hydrothermal route	300	19.4	4.4/23.2	[1]
SnO_2_ hollow microspheres	One-step calcined method	320	31.2	5.2/8.3	[2]
SnO_2_ nanoflowers	Hydrothermal method	240	6.9	25/60	[3]
SnO_2_ microstructure	Hydrothermal method	300	10	6/52	[4]
SnO_2_ nanorods	Hydrothermal route	300	13.8	5/60	[5]
SnO_2_ nanoflowers	Hydrothermal method	270	38.7	5/8	This work

#### Gas-Sensing Behaviors of Fabricated Sensors to H_2_S

**Fig. 7:**
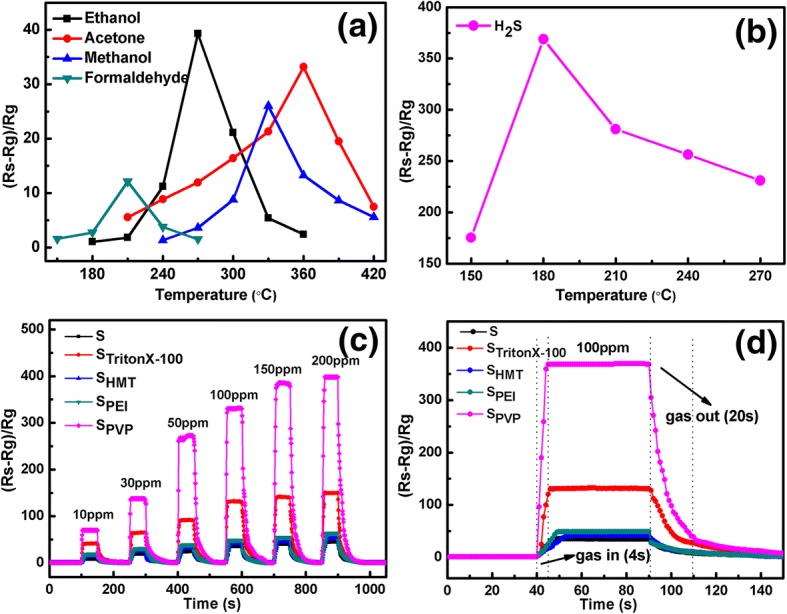
**a** Response of S_PVP_ sensor to 100 ppm ethanol, acetone, methanol, and formaldehyde at different operating temperatures. **b** Response of S_PVP_ sensor to 100 ppm H_2_S at different operating temperatures. **c** Response versus time curves of the sensors to 10–200 ppm H_2_S consecutively at180 °C. **d** Dynamic sensing transient of the sensors to 100 ppm H_2_S at 180 °C

As discussed in the previous subsection, S_PVP_ sensor exhibits the best gas-sensing property to 100 ppm ethanol owing to its high porosity. In order to find out its optimum detecting gas, we test the response of S_PVP_ sensor toward different gases, including acetone, methanol, formaldehyde, and H_2_S, with a concentration of 100 ppm at various operating temperatures (as shown in Fig. 7a, b). It can be noted that the optimal response appears at 330 °C of methanol, at 210 °C of formaldehyde, at 360 °C of acetone, and at 180 °C of H_2_S. Furthermore, the maximum response value of S_PVP_ to H_2_S is estimated to be 368, which is one or two orders of magnitude (${\mathrm{S}}_{{\mathrm{H}}_2\mathrm{S}}/{\mathrm{S}}_{\mathrm{ethanol}}=9$, ${\mathrm{S}}_{{\mathrm{H}}_2\mathrm{S}}/{\mathrm{S}}_{\mathrm{formaldehyde}}=45$) higher than that to other test gases. The lowest optimal work temperature as well as the best response value indicates S_PVP_ has the excellent selectivity to H_2_S.

Considering the high response of S_PVP_ sensor to H_2_S, we also performed a systematic gas-sensing measurement of all other sensors. A dynamic gas-sensing response and recovery curves of fabricated sensors toward H_2_S at 180 °C are displayed in Fig. 7c. Obviously, the response values of all fabricated sensors show a monotonic increasing function of H_2_S concentration. For 100 ppm H_2_S, the response and recovery time are about 9 s and 43 s for S_0_, 5 s and 30 s for S_TritonX-100_, 14 s and 40 s for S_HMT_, 8 s and 38 s for S_PEI_, and 4 s and 20 s for S_PVP_, while the maximum response values are 35, 132, 41, 49, and 368 for S_0_, S_TritonX-100_, S_HMT_, S_PEI_, and S_PVP_, respectively. It is obvious that the S_PVP_ sensor has the best response/recovery characteristics and the highest response to H_2_S gas compared with other sensors, while S_TritonX-100_ achieves the second.

**Fig. 8:**
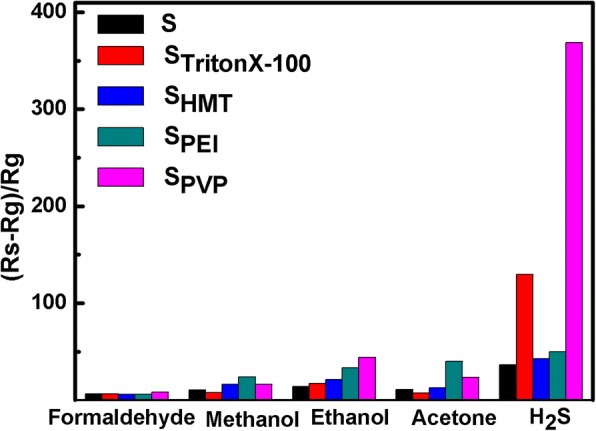
The comparison among sensor response of SnO_2_ nanomaterials to 100 ppm of various gases at the optimal operating temperature

Figure 8 displays the bar graph of response of five fabricated sensors to formaldehyde, methanol, ethanol, acetone, and H_2_S. All of the gases were tested with a concentration of 100 ppm at the optimal operating temperature. S_TritonX-100_ and S_PVP_ show a distinct response to H_2_S, while S_PEI_ shows the highest gas response to methanol and acetone. It should be mentioned that the specific surface area and the porosity are two important factors for gas sensors. The larger specific surface area will provide more active sites for adsorption and desorption of test gases, while the larger porosity would induce a greater speed of gas diffusion owing to the presence of mesopores. In comparison, S_PEI_ possesses a relative larger specific surface area than others (seen in Table 1), which shows the highest gas response to methanol and acetone (Fig. 8), while S_PVP_ and S_TritonX-100_ exhibit the higher gas response to H_2_S due to their polyporous flower-like nanostructures, proving good selectivity of S_TritonX-100_ and S_PVP_ toward H_2_S. The good selectivity of the samples to H_2_S can be explained as follows: when the SnO_2_ sensor is exposed in H_2_S gas, both chemisorbed oxygen species and SnO_2_ nanostructure react with H_2_S during sensing measurement to form SO_2_ and SnS_2_, respectively. Compared with SnO_2_, the body resistance of SnS_2_ is relatively smaller, leading to the sensitivity enhancement of the gas sensor [39]. On the contrary, the SnO_2_ sensor does not react with any other target gases, such as formaldehyde, methanol, ethanol, and acetone.

**Fig. 9:**
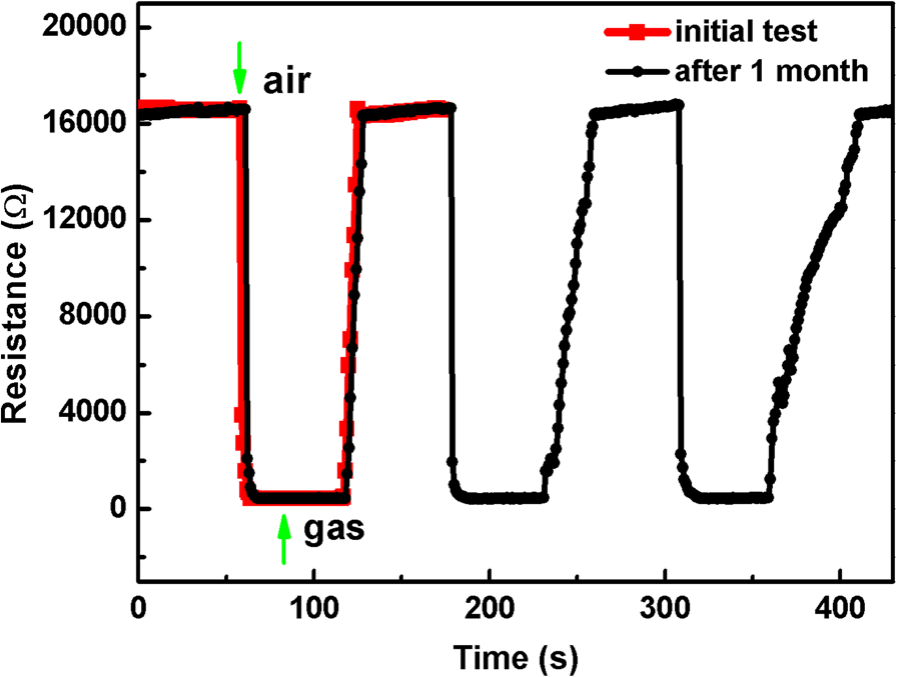
Stability of S_PVP_ over 1 month of aging for 100 ppm ethanol at 270 °C

Good stability and long service duration are expected from the viewpoint of practical application. To verify the stability of the sensor, the successive gas-sensing behavior of S_PVP_ to 100 ppm ethanol was tested under the same conditions after 1 month. The samples were stored in the vacuum drying vessel during the 1-month interval. It can be seen from Fig. 9 that S_PVP_ exhibits an excellent repeatability and stability even after 1 month. The three cyclic curves are similar to that measured 1 month ago, including the response value as well as the response-recovery time.

### Gas-Sensing Mechanism

**Fig. 10:**
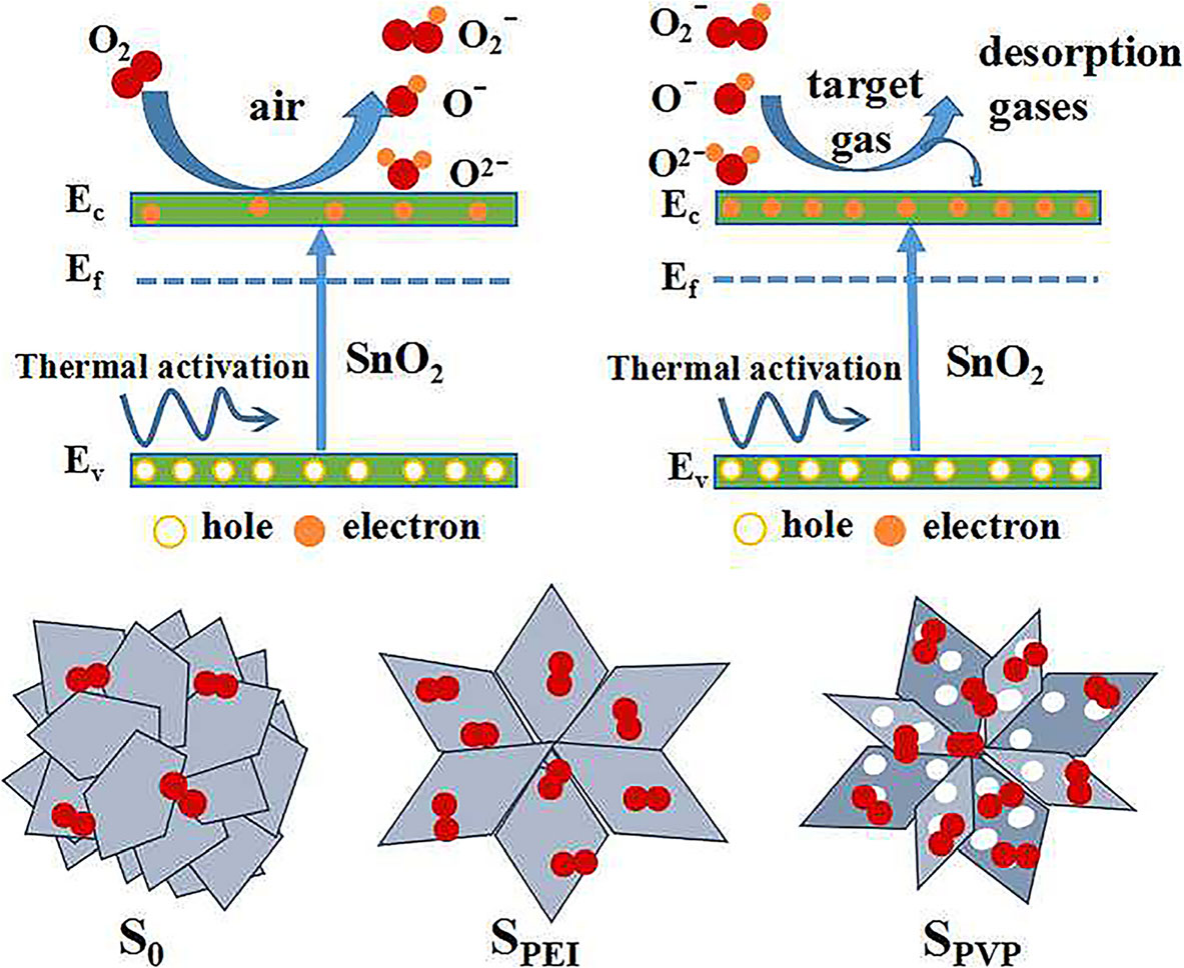
Schematic diagrams on the gas-sensing mechanism of flower-like SnO_2_ hierarchical nanostructures

Up to now, the most widely accepted gas-sensing mechanism of semiconductor oxide is the model based on the electron transfer dynamics during an adsorption–oxidation–desorption process, which can change the resistance value of the sensors [40]. The response of typical n-type semiconductor greatly depends on the electron concentration. As shown in Fig. 10, at elevated temperature, electrons in the valence band are thermally excited to the conductive band. Once the SnO_2_ sensor is exposed to ambient air, oxygen molecules will be chemisorbed on the surface of SnO_2_ nanoflowers. Oxygen ions (O_2_^**−**^, O^**−**^**,** and O^2**−**^) are then formed by capturing electrons from the conductive band of SnO_2_ [41], which is accompanied by an effective enlargement of electron-depleted layer. As a typical n-type semiconductor, the broadening of electron-depleted region means the decrease of carrier concentration within SnO_2_ nanoflowers, which will lead to the increase of resistance of the sensors. Conversely, when the SnO_2_ sensor is exposed in the reductive ambient, the absorbed oxygen species will quickly react with the target gas, which results in releasing the trapped electrons back to the conduction band and a reduction of the resistance of the sensors. Among the sensors fabricated in this work, S_PEI_ and S_PVP_ show relative better gas-sensing performances. The underlying physical mechanisms are as follows: the gas sensing properties are strongly dependent on the surface special area and the porosity. In comparison, S_PEI_ possesses a relative larger specific surface area than others, which will provide more active sites for adsorption and desorption of test gases. S_PVP_ exhibits a relative higher porosity due to the polyporous flower-like nanostructures, which is favorable to the rapid diffusion of gas (as shown in Fig. 10).

## Conclusions

We have successfully prepared the 3D hierarchical flower-like SnO_2_ nanostructures through a simple and low-cost facile hydrothermal route with the assistance of different surfactants. The images of SEM and TEM showed that the fabricated 3D hierarchical SnO_2_ nanoflowers with an average diameter of 2~4 μm were composed of many 2D nanosheets. The addition of surfactant plays an important role in the formation of nanoflowers. Based on the experimental observations, the possible growth process and gas-sensing mechanism of SnO_2_ nanoflowers were proposed. As a cationic surfactant, the addition of PEI is conducive to the nucleation of SnO_2_ nanocrystals as well as the orderly growth of SnO_2_ nanosheets, leading to a relative larger specific surface area. As amphiphilic non-ionic surfactants, PVP and TritonX-100 can make the nanosheets grow more uniformly and separately, which can serve as a soft template in the synthesis of advanced material, especially in the fabrication of mesoporous materials. In comparison, the sensor with the help of PVP (S_PVP_) exhibits excellent gas-sensing performances to ethanol and H_2_S due to its relative higher porosity. Especially, S_PVP_ shows a high response (368), fast response/recovery time (4 s/20 s), and good selectivity toward H_2_S gas. In addition, it is found that NaOH and sodium citrate are also important for the morphological formation of SnO_2_ nanoflowers.

## Abbreviations

1D: One-dimensional
2D: Two-dimensional
3D: Three-dimensional
BET: Brunauer-Emmett-Teller
FETEM: Field emission transmission electron microscopy
HMT: Hexamethylene tetramine
Na_3_C_6_H_5_O_7_·2H_2_O: Trisodium citrate dihydrate
PEI: Polyethyleneimine
PVP: Polyvinylpyrrolidone
SAED: Selected area electron diffraction
SEM: Scanning electron microscopy
XRD: X-ray diffraction
